# Exploring Teamwork Challenges Perceived by International Medical Graduates in Emergency Medicine Residency

**DOI:** 10.5811/westjem.2022.11.58002

**Published:** 2023-01-11

**Authors:** Danya Khoujah, Ahmed Ibrahim

**Affiliations:** * Tampa AdventHealth, Emergency Medicine, Tampa, Florida; † University of Maryland School of Medicine, Department of Emergency Medicine, Baltimore, Maryland; ‡ Johns Hopkins University, School of Education, Baltimore, Maryland

## Abstract

**Introduction:**

Non-US international medical graduates (IMG) represent a gradually increasing portion of emergency medicine (EM) residents in the United States. Yet there are no previous studies that explore the needs of this learner population. We conducted a qualitative study to examine non-US IMGs’ perceptions of challenges they face specifically regarding team dynamics during their first year of an EM residency.

**Method:**

Nine non-US IMGs in EM from all over the US participated in anonymous, semi-structured phone interviews lasting 45–60 minutes. We then coded and analyzed the interviews to identify axes and themes using an inductive approach informed by grounded theory. Focused coding and member checking were employed.

**Results:**

Non-US IMGs’ perceptions of challenges regarding team dynamics during their first year of an EM residency coalesced into two themes: system-based challenges, such as a new power dynamic and understanding the local hospital system, and interpersonal challenges, such as establishing rapport and articulation of critical thinking.

**Conclusion:**

Non-US IMGs perceived several unique challenges regarding team dynamics during their first year of an EM residency, whether system-based or interpersonal-based. We propose solutions such as a transitional curriculum (as suggested by the participants as well) and cultural-competence training for academic leadership.

## INTRODUCTION

Over 4,000 residents accepted through the National Resident Matching Program are non-US international medical graduates (IMG),^1^ a number that has been consistently increasing over the past two decades.^2^ This learner population forms 25% of physicians practicing in the US^3^ and a significant proportion of the healthcare services provided to the underserved.^4^ However, over half of US residency programs never interview or rank non-US IMGs,^5^ which may be attributed to unsubstantiated concerns regarding the ability of IMGs to succeed in residency.^6^,^7^ Two distinct groups of IMGs exist, all of whom completed undergraduate medical education outside the US and Canada: those who are US citizens, termed US IMGs, and those who are not, termed non-US IMGs. The two groups differ in their cultural backgrounds, prior schooling, patterns of acceptance into residency and, therefore, perceived challenges during residency. This study focused on the second group.

Prior research has identified challenges facing IMGs, such as communication, power dynamics within the healthcare team, rapport-building, and understanding the hospital system.^8^–^16^ However, none of these studies specifically addressed IMGs in emergency medicine (EM), despite the doubling of non-US IMGs matching annually in EM between 2017–2022, from 20 to 45 per year.^1^,^17^

Emergency medicine residents are evaluated according to the Accreditation Council for Graduate Medical Education Milestones in EM,^18^ which include interpersonal and communication skills that are integral to teamwork and interprofessional collaborative practice.^19^ Given the multidisciplinary approach to care in EM,^20^,^21^ understanding IMGs’ experience with team dynamics provides an important insight into their learning environment, which affects learners’ behaviors and educational outcomes.^22^ This is especially important in light of previously identified team-based challenges faced by IMGs, such as giving and receiving feedback, understanding the responsibilities of team members, team hierarchy, and addressing conflict.^9^,^14^–^16^,^23^,^24^

In this study we aimed to explore the perceived challenges related to team dynamics for non-US IMGs during their first year of an EM residency, using the qualitative methodology most suited to explore and contextualize individuals’ experiences.^25^

## METHODS

### Setting

We identified participants by manually searching EM residency websites and contacting residency program leadership, either directly or through the Council of Residency Directors in EM listserv. The invitation email described the study purpose including assurance of anonymity and offered a $20 gift card as an appreciation. Fourteen potential participants contacted the study team, and thematic saturation was achieved after nine interviews. The study was determined to be exempt by the institutional review board.

### Study Design

We used a descriptive case study design^26^ to gain an in-depth understanding of the perceived challenges. Interviews were conducted by the lead author (DK), an IMG emergency physician, from May–July 2019 to preserve consistency. The questions focused on team dynamics and were crafted by both authors from prior IMG studies in other disciplines (Table 1, Appendix 1).^8^–^10^

**Population Health Research Capsule**

What do we already know about this issue?

*Prior studies identified challenges facing international medical graduates (IMG), such as communication and team hierarchy. None has specifically addressed learners in emergency medicine.*

What was the research question?

*What are the perceived challenges related to team dynamics for non-US international medical graduates during their first year of an emergency medicine residency?*

What was the major finding of the study?

*IMGs perceive system-based (e.g., power dynamic) and interpersonal challenges (e.g., rapport) during the first year of residency.*

How does this improve population health?

*Understanding of the diversity of learners creates a more culturally competent work environment for all residents and faculty.*

### Data Collection

Participants completed an electronic consent and demographic form and were identified using a unique ID to preserve anonymity. We collected data using semi-structured phone interviews that lasted 45–60 minutes, starting with the same questions for all participants, followed by probing and clarifying questions. Some questions approached the same topic from different angles, triangulating the findings and improving the credibility of the resulting data. Iterative questioning of data with apparent conflict allowed further exploration. Interviewees’ honesty was enhanced by anonymity and voluntary enrollment. Trustworthiness was established by voluntary member-checking; interview participants reviewed summaries and themes of their own interviews for accuracy and elaborated on unclear or seemingly conflicting data.

### Data Analysis

We audio-recorded and transcribed the interviews, and then coded them using MAXQDA qualitative analysis software version 2018.2 (Verbi Software GmbH. Berlin, Germany) to organize emergent categories and their interrelationships using grounded theory methodology. DK conducted open coding and then organized codes into themes. Trustworthiness of findings was increased using focused coding; DK then partnered with an independent coder and, using the initial thematic categories, reviewed a subset of the transcripts to check for consistency and identify any overlooked themes. Disagreements were resolved through discussion. The themes were compared with known literature, and if new themes were identified, then further information was deduced from the interviews. Reflective memoing allowed the development of progressive subjectivity. Reflexivity was maintained by disclosing the researchers’ background and acknowledging its effect in knowledge construction.^25^ We did the coding inductively, avoiding quick conclusions influenced by our views. The conceptual model was built on actual quotes rather than our interpretations of quotes.

## RESULTS

### Participant Description

Nine participants were included in the study and were well-matched with average non-US IMGs, except for gender (Table 2). Approximately 90% of the sample were male, in comparison to 63.2% of non-US IMGs in EM programs.^27^ Seven participants were between the ages of 26–30, which is the same as the general demographic of EM residents and similar to the mean age of postgraduate year-1 IMGs (30.6 years).^28^ None of the participants were from residencies in the Western US, which was expected as there was only one non-US IMG accepted to a residency from that region in the 2018–2019 match.^29^ All participants were non-US IMGs who completed the first year of an EM residency between July 2016–July 2019. None had completed any residency training in North America prior to that.

Two broad themes emerged of IMGs’ perceptions of team dynamic challenges: system-based and interpersonal. Unsolicited solutions were volunteered by several participants and are included in the discussion. We edited quotes to promote clarity, and each speaker’s voice was retained as much as possible while preserving anonymity. Subthemes and exemplar quotes are summarized in Tables 3 and 4 and Appendix 2.

### Theme 1: System-based Challenges

#### Team Structure

Although most participants had a basic understanding of the concept of teamwork, a few limited their definitions to patient resuscitation-related instances. Several participants defined team members as exclusive to only physicians, while others had a more inclusive definition. One participant verbalized the lack of familiarity with the roles of different team members as these roles were not present in their home countries. Several participants described a hierarchical power dynamic between attending physicians and trainees in their previous experience, with one participant likening it to the “military.” This power dynamic differed from IMGs’ current residencies. While viewed positively by several participants, the change in hierarchy led to confusion about the expected level of formality in interactions between team members and correctly framing learning interactions.

In their home countries, a few participants had practiced within teams with authoritative rather than collaborative physician leadership where nurses were not involved in decision-making. Some participants expressed the positive effect that horizonal systems had on patient care in their new environment.

#### Prior Exposure to Emergency Medicine and Medicine in the US

The specialty of EM is not well-developed in many countries, affecting the participants’ prior exposure to it, especially given the vast difference between practicing in the ED and on the wards. Lack of familiarity with the demands of clinical work in EM may contribute to feeling overwhelmed due to the workload, busyness, and general chaos. Going in with the right mindset and expecting a high workload was helpful for one participant in tempering expectations. Having prior EM experience in the US or having worked as a research assistant eased the transition as well.

One participant commented that they didn’t know *“where patients go after they’re discharged or where they are coming from”* in reference to long-term care facilities, as these were not options they had been exposed to before.

#### Understanding the Local Hospital System

Participants used “new environment” to refer to the culture of the US as well as the hospital itself. Several participants commented on how adjusting to “the way of doing medicine” and “missing the algorithm” detracted from their educational experience. One participant commented on how US graduates have a “home-field advantage” and how that made the participant appear clinically less competent, when the issue was logistics rather than knowledge. Another participant commented that several residents in their class went to the same medical school as well, so *“they knew their way around the ER.”* In EM, understanding the logistics may be essential given the fast-paced approach.

#### Residents’ Rights and Addressing Conflict

Participants described varying levels of feeling empowered and validated; one participant felt respected and valued, whereas another shared an overt instance of “intimidation” and “injustice.” When issues such as conflict or abuse occurred, they were often not addressed due to several reasons, including lack of recognition of abuse, lack of empowerment, feeling “overwhelmed,” thinking the effort would be futile, or simply not knowing how to navigate the system. Consequently, this reflected on participants’ anticipation of fair treatment. None of the participants stated that they were explicitly denied any rights.

### Theme 2: Interpersonal Challenges

#### Establishing Rapport

The lack of “shared background” made establishing rapport difficult for several participants. One participant explained how these *“cultural differences”* made it *“hard”* to connect with others outside the workplace. One participant attributed this to *“cultural bias… they already have their own thoughts about you.”* The difficulty establishing rapport contributed to a sense of isolation (despite some having immediate family with them), as well as affecting clinical work and the ability to give feedback.

#### Cultural Competence

Culturally appropriate behaviors at participants’ home countries were sometimes viewed negatively in their new environment, specifically body language such as “gesturing,” or being “loud and boisterous.” A participant was told by an attending physician to *“stop being an aggressive (race/ethnicity)”* in reference to this demeanor. For another participant, being “quiet” and “not outspoken” were viewed as passiveness and a weakness by team members. For a third, avoiding eye contact while talking to team members was viewed as “condescending” and “ignoring,” whereas it is a culturally appropriate behavior in their home country.

Projecting confidence during interactions was another challenge that faced some participants, with resulting negative feedback if that “confidence” was not outwardly projected. One participant struggled in finding the fine line for appropriate assertiveness, risking being viewed as either aggressive or reticent.

#### Feedback

Participants’ experience in receiving feedback was variable. Several didn’t feel a “personal attack.” However, one participant felt that they were “judged harshly,” while another acknowledged that “it’s for my benefit.” More than one participant clarified that they “brush off” positive feedback as they didn’t view it as helpful. One exclaimed that the feedback process is (positively) different from their prior experiences. This difference may have contributed to the initial dismissal of the feedback by another participant. One participant attributed their perceived difficulty in obtaining feedback to a lack of faculty investment in them due to their IMG status.

#### Self-expectations

Clarity of expectations is paramount in optimizing learners’ success as one participant emphasized. Emphasis on learning rather than knowledge surprised one participant, possibly reflecting what was valued in their prior education. Unfortunately, the clarity of expectations was not the case for all participants. The disconnect between perceived and actual expectations of autonomy vs dependency, when present, negatively affected the participant’s comfort, efficiency, and performance during clinical work.

Participants viewed asking for help in contrasting lights. For some participants, their “fear of failure” and not wanting to “mess up” prompted them to ask for help “a lot,” while others would avoid asking questions if possible. Participants hesitating to ask for help either perceived it as “weakness” or did not recognize the expectations of their autonomy or knowledge, and in one case, were worried about a negative perception by the residency, as their pattern of asking for help was negatively noted on evaluations. One participant had to “learn how to ask for help.” For some, the fear of showing vulnerability was exacerbated by their IMG status. On the other hand, other participants did not perceive asking for help as a weakness and felt empowered to reach out to residency leadership outside clinical hours to seek advice.

All participants verbalized prioritizing patient safety over fear of perception of lacking knowledge or being “weak,” even when they felt “unsafe” asking questions at times or if it made them feel “stressed.”

#### Communication

“Communication is probably the most important aspect of working well in a team” especially “closing the loop” and being efficient and clear. An important part of the communication is the linguistics, and while some participants might not be comfortable using English in general, the issue for some was the spoken or informal English, rather than “textbook” English.

#### Articulation of Critical Thinking and Discussion as a Way of Learning

Articulation of critical thinking was a struggle for several participants especially with an authority figure, suggesting that discussion may not be viewed by them as a way of learning. Some participants recognized the importance of supporting arguments with evidence, and for some this improved with time.

## DISCUSSION

This study explored challenges perceived by IMGs in EM as they relate to team dynamics, revealing several system-based and interpersonal challenges. Some inconsistencies emerged in participants’ experiences, which enhances the validity of the data as the participants were a diverse group and their experiences were not anticipated to be identical.^13^,^30^

System-based challenges are expected as emergency care in the US is vastly different from countries that employ a different model of care. Integration of rehabilitation facilities, nursing homes, and other community resources in medical care is not widespread, especially in collectivist societies. While these macro-level issues have been mentioned before,^10^,^11^,^31^ the challenges regarding scope of practice and nature of interactions with other specialties is unique to EM in the US and has not been previously described. These issues are accentuated with lack of familiarity with the role of other team members such as non-physician practitioners.^10^,^32^ Horizontal relationships among healthcare staff (rather than hierarchical) are commonly different from IMGs’ prior experiences,^9^,^14^ and although embracing collaboration itself did not appear to be a challenge, the sudden shift in perspective appeared to be one.

Difficulty building rapport with patients has been investigated in prior studies,^8^,^13^ and our data shows that this challenge may be present with colleagues as well. Building collegial relationships and socialization are essential elements of learning and professional growth.^33^ Strong workplace connections may also mitigate the feelings of social isolation described by some participants, which, if unaddressed, may lead to burnout.^9^,^34^,^35^

Challenges related to communication with the team resonate with prior literature,^9^,^11^ and in our study cannot be attributed to linguistics alone as two of the nine participants consider English their first language. Contributing factors may be prior educational experiences with a sole focus on hard science^8^ and a lack of a dedicated communication curriculum.^8^

Discomfort with self-advocacy and addressing conflict are in line with previously described views of hierarchy in learning and fear of questioning authority.^9^,^13^–^15^ This lack of empowerment is exacerbated by feeling overwhelmed by other demands and not being aware of support resources.^9^

Another downstream effect of perceived hierarchy is not recognizing discussion as a learning tool, combined with prior educational experiences such as large-group learning or a “banking system of education,”^36^ as previously addressed.^13^,^15^ Non-Western cultures do not place a similar emphasis on negotiation and debate,^37^ and in many cultures silence during discussions is viewed as a sign of respect.^13^ Proactively addressing these perceptions will facilitate robust clinical discussions and decrease the frustration of both faculty and learners from poorly orchestrated teaching interactions, especially given the fast-paced workflow in the ED.

A salient perception by non-US IMGs was the need to assimilate to the dominant culture and hinder self-expression to build rapport and improve evaluations. Participants felt penalized for behaviors deemed appropriate in their home culture, which is unfortunately consistent with prior reports^9^,^14^,^38^ and probably stems from the lack of cultural competence in healthcare.^39^,^40^ A specific example is the negative feedback for lack of assertiveness mentioned by our participants, which has been reported by ethnically diverse medical students,^41^ as well as female resident physicians.^42^

This study highlights the value of a transitional curriculum as alluded to by some participants as well as documented in prior literature.^23^,^43^ Our results can be used as a needs assessment for a curriculum addressing team dynamics, rather than only knowledge and procedures.^44^ Our participants shared several topics for a clearer orientation: residents’ rights; empowerment; communication; clarification of team members’ roles; and transitions of care. A shadowing component may allow IMGs to familiarize themselves with their new environment without the added stress of performing clinical work. A participant shared their positive experience with a communication workshop they attended later in residency and reflected that it could have been helpful earlier, as noted in a prior study for IMGs in a Canadian internal medicine residency.^45^ A well-rounded approach would also address faculty and staff to improve their understanding of the diversity of learners, enabling them to have open, nonjudgmental conversations, mold clinical discussions, and accurately evaluate residents’ performance, hence avoiding contrast-effect bias and creating a more culturally competent work environment, not only for IMGs but for all.

## LIMITATIONS

This study was explorative, limiting the ability to generalize the findings to all non-US IMGs in EM, especially women, given that there was only one female participant. Participants were (at the time of the interview) at different stages of training, which may have affected the accuracy of their reflections as they spoke from memory. Some issues such as those related to power dynamics and rapport-building are likely to be widespread, while critique for culturally appropriate behavior may be specific to a country or ethnicity. Our ability to discern the influence of ethnicity or country of origin is limited as participants were not queried for this information. Some interpersonal challenges perceived may be due to local, program-related factors or a specific faculty member, affecting all trainees at the site rather than only IMGs. Prior training and practice may have affected perception as some participants expressed. Some challenges may have been related to the participants having a time gap between medical school and residency,^12^ regardless of IMG status.^7^,^46^ Finally, the study is limited by the inherent fallacies of self-perception and self-selection bias.

## CONCLUSION

International medical graduates in EM perceive several system-based and interpersonal challenges regarding team dynamics. Addressing these challenges proactively through a clear orientation curriculum may smoothen the transition. Creating a culturally competent workplace necessitates that faculty and staff learn about the values and experiences of IMGs and empower the IMGs to ask for their rights, seek help, and address conflicts when they arise.

## Supplementary Information



## Figures and Tables

**Table 1:** Examples of interview questions asked of non-US international medical graduates.

All of these questions relate to your first year of residency. In your opinion:
What are some of the challenges you faced when working with your team members? What was your perception of the value of the opinions of other members in your team? What was your experience with receiving feedback from your team members in your first year of residency? What were your expectations of your role as a resident? What was your understanding of your rights as a resident? What are some behaviors that you thought your team would perceive as a weakness for a team member?

**Table 2:** Description of participants (n=9).

Descriptor	Number	Percentage
Gender		
Male	8	88.9%
Female	1	11.1%
Age		
26–30	7	77.8%
31–35	2	22.2%
Region of the US where the first year of EM residency was completed		
Northeast	3	33.3%
Midwest	1	11.1%
South	5	55.6%
Time gap between medical school graduation and start of EM residency		
1–3 years	5	55.6%
4–7 years	4	44.4%
Consider English to be their first language		
Yes	2	22.2%
No	7	77.8%

**Table 3:** Team-dynamic challenges perceived by non-US international medical graduates in emergency mnedicine residencies: system-based challenges.

Subtheme	Exemplar quotes
**Team structure**	
Team members	“We don’t have techs. I didn’t know what a CRNA was.” (Participant 1, referring to their home country)
Power dynamic	“People from – generally speaking – the Middle East, Asia, there is a different level of authority. If your attending is a little bit bossy, a little bit difficult to deal with, we just take it, it’s not a big deal.” (Participant 6)
Hierarchy between physicians and non-physicians	“It’s mainly hierarchical. The attending is supposed to be the most respected person on the team, and then you’d have your senior resident. When a nurse or a patient care technician would ask you a question, you would answer the question, but you would not be regarding their thoughts into your decision making.” (Participant 2, referring to their home country)
**Prior exposure to EM and medicine in the US**	
Current state of EM in other countries	“Emergency medicine is one thing that the US is leading, and my country is far behind in properly running the emergency department. Being familiar with how things are run in the emergency department is definitely different from how things are done in other specialties.” (Participant 8)
Nature of prior EM experience	“My expectation was that my life outside of work will be very limited and I would have long work hours and I would have only few days off per month every month.” (Participant 9)
Logistics (e.g. transitions of care)	“I never understood “this patient is appropriate for an LTCF or a skilled nursing facility.” I couldn’t grasp it. It was very abstract for me.” (Participant 1)
**Understanding the local hospital system**	
“New environment”	“I didn’t know my place. Where should I be standing exactly?” (Participant 5)
Splitting attention between acclimation and medical knowledge	“You’re coming to a new place and new space, and you look different and people know you’re different as opposed to someone who “grew up in that environment.” They’d be much more familiar with everything around them. They’re already familiar with the interactions, whether with patients or other providers, so they can focus on other things such as improving their clinical care alone as opposed to trying to improve everything; trying to acclimate yourself with everything.” (Participant 1)
**Residents’ rights and addressing conflict**	“I didn’t realize that you can express yourself and express your concern and you can fight for your rights and you can have a discussion about things that make you uncomfortable.” (Participant 4)

*CRNA*, certified registered nurse anesthetist; *LTCF*, long-term care facility; *EM*, emergency medicine.

**Table 4:** Team-dynamic challenges perceived by non-US international medical graduates in emergency medicine residencies: interpersonal challenges.

Subthemes	Exemplar quotes
Establishing rapport	“Being able to talk during the shift about things outside of clinical work. It was difficult for me.” (Participant 1) “They talk about wine a lot; I have no idea what they’re talking about.” (Participant 9) “I felt like I was alone in all of this” (Participant 6)
Cultural competence	“I had to recognize the cultural issues that I had or what I grew up with or what I have learned before and then try to modify that to make it more applicable to the culture that I’m doing my training in.” (Participant 2)
Feedback	“Americans are so nice in giving feedback … so my experience with it has been great.” (Participant 7)
Self-expectations	
Clarity	“I was like …. I have to know everything… But then you come here and everybody emphasizes that you being a good learner, accepting feedback well, having a positive attitude, asking for feedback, and being respectful are the core values, are the things that really matter rather than coming in with a knowledge base.” (Participant 7) “My residency program was not clear about my exact duties: what I should do, what I shouldn’t do.” (Participant 3)
Autonomy	“I was transferring some sort of a [home country] model. You should let the attending know what you are about to do and then they agree to it (before going ahead with the plan).” (Participant 4) “I worked in [home country] on my own in the ER for two years and I was used to making my own decisions” (Participant 3)
Asking for help	“I didn’t want to confirm somebody’s suspicions that I’m an imposter.” (Participant 1) “Even though I felt that it was my right, if I’m still asking very basic questions, they were perceiving it as my weakness (as mentioned in performance evaluations).” (Participant 8)
Vulnerability	“I felt like other people were more comfortable sharing their mishaps or their academic weaknesses because they had that background of being from here… As opposed to me, where anytime I felt like I had an academic weakness it was because I came from an outside program and therefore I was weak.” (Participant 1)
Communication	“People here communicate using a lot of American expressions that I was not accustomed to, so I would use formal English to convey a message instead of an expression that would seem more appropriate for the situation.” (Participant 7)
Articulation of critical thinking and discussion as a way of learning	“If I thought differently, I would rarely bring it up with someone more senior than me… Me sharing things did not play a role in my education.” (Participant 5) “I started to back up my opinion before even talking so I know what I’m talking about, so you can have a productive, good conversation about anything, which you learn from, which is good.” (Participant 4)

*US*, United States.
