# Supplementary material for: Exploring Teamwork Challenges Perceived by International Medical Graduates in Emergency Medicine Residency
Source: West J Emerg Med. 2023 Jan 11;24(1):50–8. doi: 10.5811/westjem.2022.11.58002 (PMC9897247; doi:10.5811/westjem.2022.11.58002)
Supplement: Supplementary file 1 [file wjem-24-50-s001.docx]

**Appendix 1: Complete List of Interview Questions**

| All of these questions relate to your first year of residency. In your opinion:   - What are some of the challenges you faced when working with your team members? - During your first year of residency, what was your understanding of teamwork? - What was your perception of the value of the opinions of other members in your team? - Can you please describe to me any challenges you may have had when you shared your opinion with someone more senior than you in your team if it’s different than yours? - What was your experience with giving feedback to your team members in your first year of residency (positive and negative)? - What was your experience with receiving feedback from your team members in your first year of residency (positive and negative)? - What were your expectations of your role as a resident (autonomy, dependence, clarity)? - What was your understanding of your rights as a resident? - What are some behaviors that you thought your team would perceive as a weakness for a team member (not coming to work when sick, not knowing how to do something)? - What are the effects of these perceptions on your stress level at work? - What did you do when you needed to ask for help in relation to clinical work? |
| --- |

**Appendix 2: Team-dynamic Challenges Perceived by Non-US IMGs in EM Residencies: System-based Challenges**

| **Subtheme** | **Exemplar Quotes** |
| --- | --- |
| **Team structure** |  |
| Team members | *“We don’t have techs. I didn’t know what a CRNA was*.” (Participant 1, referring to their home country) |
| Power dynamic | *“People from – generally speaking ­– the Middle East, Asia, there is a different level of authority. If your attending is a little bit bossy, a little bit difficult to deal with, we just take it, it’s not a big deal.” (*Participant 6)  *“They are the people in power, so I try to compromise and settle. I then realized, it’s not us vs them”* (Participant 4)  *“It’s fascinating when you see the whole team listening to a medical student presenting a complicated case, appreciating everything they say, welcoming their opinion. They appreciate everything any member of the ED says even the nursing staff, so I think it was a good environment.”* (Participant 4) |
| Hierarchy between physicians and non-physicians | *“It’s mainly hierarchical. The attending is supposed to be the most respected person on the team, and then you’d have your senior resident. When a nurse or a patient care technician would ask you a question, you would answer the question, but you would not be regarding their thoughts into your decision making.”(*Participant 2, referring to their home country)  *“You have to include your nurses in the decision making because they are the last filter before you actually make a mistake.” (*Participant 2) |
| **Prior exposure to EM and medicine in the US** |  |
| Current state of EM in other countries | *“I knew what I was getting into but it was on a different scale of craziness (in the US)”* (Participant 6)  *“Emergency medicine is one thing that the US is leading and my country is far behind in properly running the emergency department. Being familiar with how things are run in the emergency department is definitely different from how things are done in other specialties.”* (Participant 8) |
| Nature of prior EM experience | *“My expectation was that my life outside of work will be very limited and I would have long work hours and I would have only few days off per month every month.”*(Participant 9) |
| Logistics e.g. transitions of care | *“I never understood “this patient is appropriate for an LTCF or a skilled nursing facility”. I couldn’t grasp it. It was very abstract for me.”* (Participant 1) |
| **Understanding the local hospital system** |  |
| “New environment” | *“I didn’t know my place. Where should I be standing exactly?”* (Participant 5) |
| Splitting attention between acclimation and medical knowledge | *“You’re coming to a new place and new space, and you look different and people know you’re different as opposed to someone who “grew up in that environment”. They’d be much more familiar with everything around them. They’re already familiar with the interactions, whether with patients or other providers, so they can focus on other things such as improving their clinical care alone as opposed to trying to improve everything; trying to acclimate yourself with everything.”* (Participant 1) |
| **Residents’ rights and addressing conflict** | *“I knew what we (the residents) had to say was respected and mattered.”* (Participant 1)  *“I didn’t realize that you can express yourself and express your concern and you can fight for your rights and you can have a discussion about things that make you uncomfortable.”* (Participant 4)  “*During my second year there was a course about abuse and there was a questionnaire that we had to do. I was like, oh my gosh, a lot of these examples happened to me”* (Participant 6)  *“I knew my rights, I just didn’t push for them aggressively”* (Participant 1)  *“I don’t know how to address microaggression from an attending.”* (Participant 2)  *“Residents are at the bottom of the hierarchy. If something happens between a resident and a nurse, definitely the nurse is gonna be the one who is right because it’s clear to them what are the rights and to me it wasn’t. I didn’t know what I was supposed to do when it comes to that.”* (Participant 2)  *“I felt if there’s a conflict, they (the leadership) will ask me for my stance and my justification, and then make their own decision”* (Participant 8) |

*Abbreviations: EM, emergency medicine; IMGs, international medical graduates; US, United States*

**Appendix 3: Team-dynamic Challenges Perceived by Non-US IMGs in EM Residencies: Interpersonal** **Challenges**

| **Subthemes** | **Exemplar Quotes** |
| --- | --- |
| **Establishing rapport** | *“Being able to talk during the shift about things outside of clinical work. It was difficult for me.”* (Participant 1)  *“People didn’t know who I am or what I was made of… As soon as they find out you’re an IMG, it’s like “oh okay I’m not interested in this conversation any longer.” It’s a conversation ender for me.”* (Participant 1)  *“When they talk about things that happen to you in America, things that happen in school, things they do, a lot of the time I don’t have that in common with them so I don’t have anything to say about the subject. Sometimes I don’t understand exactly what they’re talking about. For example, they talk about wine a lot; I have no idea what they’re talking about.”* (Participant 9)  *“I didn’t have anyone that I could connect with and share these issues with.”* (Participant 1)  *“I felt like I was alone in all of this”* (Participant 6)  *“Because I was international, people were more likely to discount my opinion and sometimes with particular team members it would be a direct “You’re just saying that because you’re from X.” That was difficult”* (Participant 1)  *“People didn’t know who you are and they have to know you first to trust you. If they didn’t know you enough, giving them feedback or trying to explain something in terms of patient care would be taken in a negative way.”* (Participant 2)  *“I felt that people wouldn’t take it seriously. Not coming from a US medical school, I didn’t feel like I had the “rep”, the “cred”, to be able to do that. It’s like “what do I know.””They’d be like “yeah probably everything for you is impressive”.”* (Participant 1, regarding them giving a team member positive feedback)  *“I did not feel I had the right to give anybody feedback. No matter if that is correct or not, but that’s how I felt.”(* Participant 5) |
| **Cultural competence** | *“I had to recognize the cultural issues that I had or what I grew up with or what I have learned before and then try to modify that to make it more applicable to the culture that I’m doing my training in.”* (Participant 2) |
| **Feedback** | *“Americans are so nice in giving feedback and they have studied the art of giving feedback. It’s a very diplomatic, gentle, and nice process that is very constructive, so my experience with it has been great.”* (Participant 7)  *“Sometimes people give you some hints and I was like this is tiny feedback, I’m going to consider it, but then as I progressed, I realized it’s a big, big problem.”* (Participant 4)  “*I really wanted feedback and I felt like it was hard to find a mentor that would be invested in me as a person. I felt like I don’t share a common background with them - I didn’t feel like someone would have a vested interest in me.*” (Participant 1) |
| **Self-expectations** |  |
| Clarity | *“I was like oh my God I have to know everything. I have to perform. I have to know my medicine. But then you come here and everybody emphasizes that you being a good learner, accepting feedback well, having a positive attitude, asking for feedback, and being respectful are the core values, are the things that really matter rather than coming in with a knowledge base.”* (Participant 7)  *“My residency program was not clear about my exact duties: what I should do, what I shouldn’t do. It was like “yeah go ahead and see patients.” The image was not clear to me.”* (Participant 3) |
| Autonomy | *“I was transferring some sort of a [home country] model. You should let the attending know what you are about to do and then they agree to it (before going ahead with the plan). Here in the US, you can start some basic stuff without talking to anybody and they can adjust it.”(*Participant 4)  *“I worked in [home country] on my own in the ER for two years and I was used to making my own decisions”* (Participant 3) |
| Asking for help | *“I would blame myself if I didn’t know things as opposed to being okay with asking about them.”* (Participant 6)  *“I should really toughen up. Nobody really said that but I felt that was the expectation.”* (Participant 6)  *“if I am asked to do something I should go and do it, if I involve someone with me it will be a problem”* (Participant 4)  *“I didn’t want to confirm somebody’s suspicions that I’m an imposter.”* (Participant 1)  *“Even though I felt that it was my right, if I’m still asking very basic questions, they were perceiving it as my weakness* (as mentioned in performance evaluations)*.”* (Participant 8)  *“I realized late that people have a fantastic point of view of things that you can implement really quickly just by listening to them, specifically if it’s someone who has a good experience or a good clinician. Maybe if I started talking to them early during my career, maybe I would solve these problems quicker.”* (Participant 4) |
| Vulnerability | *“I felt like other people were more comfortable sharing their mishaps or their academic weaknesses because they had that background of being from here, “oh, you went to a prestigious American medical school so you can afford to not know something.” As opposed to me, where anytime I felt like I had an academic weakness it was because I came from an outside program and therefore I was weak.”(*Participant 1) |
| **Communication** | *“Sometimes I appreciate what someone says and I don’t give them feedback immediately. I adjusted this behavior which improved my communication and their perception (by saying) ”I appreciate what you said.” I learned that this is extremely important here in the US culture.”* (Participant 4)  *“People here communicate using a lot of American expressions that I was not accustomed to, so I would use formal English to convey a message instead of an expression that would seem more appropriate for the situation.”* (Participant 7) |
| **Articulation of critical thinking and discussion as a way of learning** | *“If I thought differently, I would rarely bring it up with someone more senior than me. Not necessarily because I was afraid I would be wrong, but because I felt I should just be learning and this is what I should be doing next. They are right and mine is just an opinion. Me sharing things did not play a role in my education.”* (Participant 5)  *“Certain attendings sometimes disagree with me and I feel frustrated, like the attending is against me, so I tell them okay I’ll do whatever you want, so they thought that I’m not confident enough.”* (Participant 3)  *“I started to back up my opinion before even talking so I know what I’m talking about, so you can have a productive, good conversation about anything, which you learn from, which is good.”* (Participant 4) |

*Abbreviations: EM, emergency medicine; IMGs, international medical graduates; US, United States*
